# Dynamic Changes and Correlations of Physicochemical Parameters, Flavor Compounds and Microbial Communities During Soy Sauce Koji Production

**DOI:** 10.3390/foods15122133

**Published:** 2026-06-13

**Authors:** Ziwei Liu, Guangsen Fan, Huanlu Song, Xiaoyan Liu, Rifeng Chen, Zhili Yu, Jiang Yu

**Affiliations:** 1 Key Laboratory of Geriatric Nutrition and Health (Beijing Technology and Business University), Ministry of Education, Beijing 100048, China; 18333260154@163.com (Z.L.); liuxiaoyan@btbu.edu.cn (X.L.); 2 School of Food and Health, Beijing Technology and Business University, Beijing 100048, China; 3 Shandong Luhua Biotechnology Co., Ltd., Laiyang 265200, China; chrifeng@126.com; 4 School of Science, Xi’an Jiaotong-Liverpool University, Suzhou 215123, China; zhili.yu22@student.xjtlu.edu.cn

**Keywords:** soy sauce koji production, physical and chemical properties, volatile flavor compounds, microbial diversity, correlation network

## Abstract

Koji production is a critical process that determines the flavor and quality of the final soy sauce product. However, the complex mechanisms underlying microbial metabolism and the evolution of the physicochemical environment still require further analysis. This study focuses on three parallel koji rooms in an industrialized koji fermentation process. This work tracked the dynamics of physicochemical indices, volatile flavor compounds, and microbial communities over a full 40 h cycle. Data integration and correlation analysis elucidated the close linkage between the microbial community, the fermentation environment, and flavor formation. Koji moisture declined gradually, with faster losses at later fermentation stages. This physiological dehydration arose from microbial metabolic heat, forced aeration and structural loosening of koji, not simple physical evaporation. System pH displayed a typical U-shaped trend across fermentation. Values dropped early, most likely driven by accumulating organic acids, before rising from mid to late fermentation. This pH rebound was tentatively attributed to ammonia release from proteolytic breakdown, which may neutralize acidic compounds. These observations cast doubt on the conventional assumption that organic acid levels may be reliably estimated solely from pH measurements. Physicochemical analysis showed continuous accumulation of amino acid nitrogen (0.6–0.9 g/100 g) and total acidity throughout fermentation. By contrast, reducing sugar concentrations differed across individual koji rooms, presumably owing to divergent microbial adaptation in early fermentation. A total of 77 common compounds were identified, among which 13 key odor-active compounds with OAV ≥ 1, such as 4-vinylguaiacol and 3-methylbutyraldehyde, constitute the characteristic flavor profile of soy sauce starter culture. High-throughput sequencing uncovered a distinct ecological pattern: eukaryotic communities, dominated by *Aspergillus oryzae*, converged under controlled regulation. While prokaryotic communities differentiated dynamically, driven by spatial heterogeneity in the semi-open fermentation environment. Spearman correlation analysis further indicated potential functional partitioning: high-abundance taxa (e.g., *Aspergillus oryzae*, *Weissella*) were predominantly associated with macromolecular substrate degradation, whereas rare low-abundance taxa (e.g., *Alternaria*) displayed significant correlations with the biosynthesis of key characteristic flavor compounds. This study clarifies the synergistic regulatory mechanisms linking physicochemical conditions, microbial metabolism, and flavor precursor formation during industrial koji production. The findings establish a scientific foundation for optimizing process parameters and achieving standardized quality control in soy sauce manufacturing.

## 1. Introduction

Soy sauce is a traditional fermented condiment originating in Asia, whose production from soybean meal and wheat relies on a complex, microbially driven fermentation process [1,2]. Owing to its distinctive salty, savory, and rich flavor, soy sauce serves as a culinary cornerstone in many Asian cuisines, including those of China, Japan, South Korea, and Thailand. Furthermore, its global influence and market presence are steadily expanding [3,4]. The sensory and functional profile of soy sauce results from a series of microbial-driven processes. Enzymatic degradation of macromolecules is followed by metabolic conversion into end products, including organic acids, amino acids, and volatile flavor compounds, which collectively shape the final quality [5].

Fermentation process differences primarily divide commercial soy sauce into two types: high-salt liquid-state and low-salt solid-state fermentation [6]. Commercial production primarily follows two distinct processes: (1) high-salt dilute-state fermentation, which involves a long-term, low-temperature process yielding a rich, complex flavor. (2) low-salt solid-state fermentation, a shorter-cycle process that prioritizes efficiency and cost control, resulting in a milder product [7]. These methods, respectively, satisfy different market segments. Soy sauce brewing is a sequential, synergistic bioprocess encompassing three core stages: koji production, moromi fermentation, and pressing/sterilization. Koji production constitutes the foundational and pivotal control point for the entire process [8]. Industrial koji production traditionally relies on inoculating steamed grains with a pure *Aspergillus oryzae* culture to synthesize the hydrolytic enzymes (proteases, amylases) essential for moromi fermentation. Nevertheless, due to the semi-open nature of the process, a diverse array of environmental microorganisms naturally colonizes the substrate. These indigenous microbes undergo succession alongside the inoculated mold, collectively defining the foundational microbial consortium and its metabolic potential. Although manufacturers adopt similar production procedures, inherent ecological complexity largely accounts for the differences in product quality and flavor characteristics [9,10].

To understand the structure and dynamics of microbial communities in the complex soy sauce fermentation system, researchers have developed a diverse array of methodologies. Early traditional microbiological methods based on culture laid the foundation for functional studies of brewing microorganisms. A significant breakthrough was achieved with the introduction of molecular fingerprinting techniques, such as DGGE, TGGE, T-RFLP, and RAPD analysis [11,12,13]. These methods transcended the constraints of traditional culturing, vastly deepening our insight into the microbial diversity present in soy sauce fermentation systems. The rapid development of high-throughput sequencing and multi-omics technologies now allows for high-resolution, systematic profiling of microbial communities in complex fermentations. This capability to analyze structure, succession, and functional potential in real-time has cemented their pivotal role in research on soy sauce microbiota and quality development.

While it is well-established that microbial succession during koji production critically determines final soy sauce quality, significant knowledge gaps persist. Furthermore, a notable disconnect remains between existing scientific understanding and practical industrial application. First, existing research has largely focused on laboratory-simulated koji production systems or small-scale analyses of individual koji rooms. There is a severe lack of systematic studies examining the synchrony of microbial community succession, consistency in physicochemical metabolism, and patterns of flavor precursor formation across parallel koji rooms within the same production batch during industrial-scale production. The production stability of parallel koji rooms is a core industrial bottleneck that constrains the standardization of the koji production process and the uniformity of soy sauce product quality. Secondly, existing studies have often treated the microbial community, physicochemical parameters, and flavor compounds of koji in isolation, lacking a systems-level perspective on their dynamic interactions throughout production. Consequently, the proposed intrinsic link between microbial activity and the formation of metabolites and flavor precursors in industrial systems thus remains inadequately supported by empirical process data. Third, existing research has not yet fully elucidated the patterns of dynamic succession of non-inoculated associated microorganisms in semi-open koji production systems, nor their contributions to metabolic activity and flavor development during the koji production process [14].

To address the aforementioned scientific issues and industrial bottlenecks, this study examined three parallel koji production rooms from the same production batch at Shandong Luhua Soy Sauce Factory. Systematic dynamic sampling was conducted throughout the entire koji production cycle. Key physicochemical indices were tracked throughout koji fermentation, and volatile flavor profiles were determined via GC-MS. High-throughput sequencing was further applied to characterize the diversity, composition and succession of microbial communities. On this basis, the study systematically elucidates the intrinsic relationship among microbial community succession, changes in physicochemical parameters, and the formation of flavor compounds during the industrialized koji production process. Two core novelties differentiate the present investigation from earlier studies on koji fermentation. First, comparative analysis across independent industrial koji rooms under semi-open production conditions uncovered real-world inter-room variations in physicochemical properties and microbiota, which cannot be fully reflected by laboratory-scale controlled fermentation. Second, parallel fermentation tests revealed divergent succession features: fungal communities tended to converge while bacterial communities varied distinctly, implying fungi possess stronger environmental stability, whereas bacteria are more susceptible to subtle microenvironmental shifts. Collectively, these observations enrich the mechanistic understanding of microbial regulation behind quality fluctuation and consistency during industrial koji production. The findings of this study aim to elucidate the microbiological mechanisms underlying the formation of soy sauce quality during industrialized koji production, thereby providing a solid scientific basis and theoretical foundation for the standardization and optimization of production processes, as well as for the consistent improvement and stabilization of product quality.

## 2. Materials and Methods

### 2.1. Materials and Reagents

The soy sauce koji used in this study was provided by Shandong Luhua Co., Ltd. (Laiyang, China) For systematic profiling of microbial succession over industrial koji fermentation, three independent koji rooms from a single production batch were set as biological replicates and sampled sequentially across the whole fermentation period. Sampling was performed at 6 h, 12 h, 20 h, and 40 h throughout koji fermentation to cover key physiological and biochemical stages of the manufacturing process. Representative composite samples are obtained via the five-point sampling method to guarantee sample homogeneity. All collected samples are divided into two portions. One portion is temporarily stored at 4 °C for the determination of physicochemical indices and flavor compounds, while the other is preserved at −80 °C for subsequent microbial community analysis.

### 2.2. Methods

#### 2.2.1. High-Throughput Sequencing of Microbial Communities

Microbial community high-throughput sequencing was performed by Shanghai Meiji Biotechnology Co., Ltd., Shanghai, China. Total microbial genomic DNA was extracted with a commercial DNA extraction kit, and DNA integrity was verified using 1% agarose gel electrophoresis. Using total genomic DNA as the template, the V3–V4 region of the bacterial 16S rRNA gene is amplified via PCR with the primer pair 338F (5′-ACTCCTACGGGAGGCAGCAG-3′) and 806R (5′-GGACTACHVGGGTWTCTAAT-3′). The fungal ITS1–ITS2 region is amplified using the primer set ITS1F (5′-CTTGGTCATTTAGAGGAAGTAA-3′) and ITS2R (5′-GCTGCGTTCTTCATCGATGC-3′). After verification via 2% agarose gel electrophoresis, the amplification products were purified using the DNA gel kit (Axygen Biosciences, Union City, CA, USA), quantified and used to prepare a library with the TruSeq™ DNA Sample Prep Kit (Illumina Inc., San Diego, CA, USA). Paired-end sequencing was conducted using the Illumina NextSeq 2000 platform to generate raw sequencing data for microbial communities. Taxonomic classification of representative Operational Taxonomic Unit (OTU) sequences was performed against the SILVA v138 database (for bacteria) and the UNITE v8.3 database (for fungi).

#### 2.2.2. Determination of Physical and Chemical Properties

All physicochemical indices are determined in triplicate, and results are expressed as the mean ± standard deviation. Detailed measurement procedures are provided below. The pH value is determined based on the method described by Qi et al. [15] with minor modifications. Briefly, 5.0 g of the koji sample is weighed and homogenized with distilled water, and the solution volume is adjusted to 250 mL. After standing for 30 min, take 20 mL of the supernatant and measure the pH directly using a pH meter.

Moisture content is measured according to the direct drying method described in the national food safety standard GB 5009.3—2016 [16]. Briefly, 5.0 g of sample (accurate to 0.0001 g) is placed in a weighing bottle and dried to a constant weight. The sample is dried at 105 °C for 4 h, cooled for 30 min, and weighed. Subsequent drying is performed for 1 h, followed by another 30 min cooling and weighing procedure. This process is repeated until the weight difference between two consecutive measurements is less than 0.002 g.

Total acid and amino acid nitrogen contents are determined via acid–base titration in accordance with the national food safety standard GB5009.235-2016 “National Food Safety Standard: Determination of Total Acid in Foods.” [17]. Weigh 5.0 g of the sample, add carbon dioxide-free water, mix thoroughly, and the mixture volume is fixed to 100 mL. Mix well, filter, and collect the clear filtrate. Take 20.0 mL of the filtrate and titrate it with a standard sodium hydroxide solution to a pH of 8.2. Record the volume of solution consumed to calculate the total acid content in the sample. Next, add 10.0 mL of formaldehyde solution to the titration system described above. After mixing, continue titrating with the standard solution until the pH reaches 9.2. The corresponding consumed volume is recorded to determine the amino acid nitrogen content.

Reducing sugar content is determined using the DNS colorimetric method reported by Yao et al. [18] with minor modifications. Anhydrous glucose dried to constant weight, a stock solution (1.0 mg/mL) was prepared and subsequently diluted to generate a series of standard solutions for constructing a calibration curve. For each standard and diluted sample, 250 μL was transferred to a centrifuge tube, followed by the addition of 250 μL of DNS reagent. After thorough mixing, the tubes were heated in a boiling water bath for 10 min and subsequently cooled. Then add 250 μL of a 40% (w/v) sodium solution of NaKC_4_H_4_O_6_·4H_2_O, mix well and measure the absorbance at a wavelength of 540 nm.

#### 2.2.3. Enzyme Activity Assay

Amylase activity was determined using the DNS method. The amount of enzyme that hydrolyzes soluble starch to produce 1 mg of maltose within 5 min at 40 °C and pH 5.6 is defined as one unit of amylase activity, expressed as U/g [19]. Add 200 μL of 1% starch substrate to the test tube. After preheating at 40 °C for 5 min, add 200 μL of appropriately diluted enzyme solution and mix thoroughly. After reacting at 40 °C for 10 min, add 400 μL of DNS reagent to stop the reaction, heat in a boiling water bath for 10 min, and then cool. Measure the absorbance using a spectrophotometer at a wavelength of 540 nm. A control group was established simultaneously, using an inactivated enzyme solution as the blank control.

Protease activity was determined using the Folin-phenol method. Enzyme activity is defined as follows: at 40 °C and the corresponding pH, 1 μg of tyrosine produced by the hydrolysis of casein in 1 min is defined as 1 unit of enzyme activity [20]. Take 100 μL of appropriately diluted enzyme solution, add 100 μL of substrate preheated to 40 °C, mix well, and incubate in a 40 °C water bath for 10 min. Immediately after removal, add 200 μL of trichloroacetic acid solution to stop the reaction. After mixing, take 100 μL of the supernatant, add 500 μL of Na_2_CO_3_ solution and 100 μL of Folin–Ciocalteu reagent, mix well, and incubate at 40 °C for 20 min. After cooling, measure the absorbance at a wavelength of 680 nm.

#### 2.2.4. Detection of Volatile Flavor Compounds

Analysis of volatile flavor compounds in samples using Headspace Solid-Phase Microextraction (HS-SPME) coupled with GC×GC-O-MS. Quantification was performed using the internal standard method (2,4,5-trimethylthiazole, 1.013 μg/μL). The extraction and instrumental analysis conditions were based on the method reported by Tang et al. [21] with appropriate modifications. For HS-SPME analysis, 3.0 g of sample was placed in a headspace vial. After equilibration at 60 °C for 20 min, a DVB/CAR/PDMS SPME fiber was exposed to the vial headspace for 40 min at 60 °C. Subsequently, the fiber was inserted into a preheated GC injector (230 °C) for thermal desorption over 6.5 min under splitless conditions.

Chromatographic conditions: The 1D column was a DB-WAX (30 m × 0.25 mm, 0.25 μm) and the 2D column was a DB-17 (1.85 m × 0.18 mm, 0.18 μm). Helium (purity ≥ 99.999%) was used as the carrier gas. The oven temperature program was: 40 °C (held for 5 min), increased at 4 °C/min to 230 °C (held for 3 min). The carrier gas flow rate was maintained at 1 mL/min in splitless mode. Mass spectrometric conditions: Electron impact (EI) ionization was performed at 70 eV. The ion source temperature was 230 °C, and the quadrupole temperature was 150 °C. Full-scan acquisition was used over a mass range of *m*/*z* 40–400.

#### 2.2.5. Determination of Free Amino Acids

Briefly, 3.0 g of the sample is diluted with ultrapure water to a final volume of 30 mL. The mixture is ultrasonically extracted at 40 °C for 30 min, followed by centrifugation at 8000 r/min for 10 min. The supernatant is filtered through a 0.22 μm membrane prior to HPLC injection.

Mixed amino acid standard stock solution is serially diluted with 0.1 mol/L HCl to obtain standard solutions with gradient concentrations. A calibration curve is established by plotting peak area against corresponding standard concentration for quantitative calculation of amino acid contents in samples.

Chromatographic conditions: Separation was performed on an Agilent Zorbax Eclipse-AAA column. Mobile phase A was a 40 mmol/L NaH_2_PO_4_ solution adjusted to pH 7.8; mobile phase B was pure methanol; mobile phase C was ultrapure water; mobile phase D was a ternary mixture of methanol, acetonitrile and water (45:45:10, V/V/V). Gradient elution was used. The flow rate was 1 mL/min, and the injection volume was 1 μL. UV detection was performed at 338 nm, and the column temperature was set at 40 °C.

### 2.3. Data Analysis

After raw data obtained from high-throughput sequencing undergoes quality control filtering, paired-end assembly, and preprocessing to remove chimeric and low-quality sequences, OTUs are clustered based on 97% sequence similarity. Alpha diversity and sequencing depth were assessed based on the clustered OTUs. Subsequently, the microbial community composition and relative abundance at each taxonomic level were analyzed according to the taxonomic annotation results. Raw effective reads of all samples ranged from 47,895 to 76,187. All samples were randomly subsampled to a uniform depth of 26,000 reads to ensure comparability of microbial community diversity and composition across samples. All of the above bioinformatics analyses were performed on the I-Sanger bioinformatics cloud analysis platform at Shanghai Meiji Biotechnology Co., Ltd. (www.i-sanger.com). Data were collated and managed using Microsoft Office Excel 2019. All statistical analyses were performed with IBM SPSS Statistics (version 24). Raw materials were uniformly blended and split equally into three separate koji rooms for fermentation, resulting in three biological replicates for each treatment. For all physicochemical and microbial detection indicators, each individual sample was analyzed in three technical parallel replicates. Quantitative data are expressed as the mean ± standard deviation (Mean ± SD). Differences among groups were assessed by one-way ANOVA, followed by Duncan’s multiple range test for post hoc comparisons. Prior to ANOVA, the Shapiro–Wilk test was used to assess data normality, while Levene’s test was applied to examine homogeneity of variances. ANOVA was performed only when both assumptions were satisfied. Where data did not meet the ANOVA prerequisites, nonparametric statistical analysis was adopted. A *p*-value of less than 0.05 was considered statistically significant. The structural identification of volatile compounds was conducted by a rigorous approach that required consistency across three independent parameters: (1) mass spectrum matching against the NIST 17 library; (2) comparison of calculated retention indices (RI) with literature values, using a C7-C40 n-alkane series for RI calibration; (3) concordance of the odor detected by GC-O with the aroma attributes described in the literature [22].

Correlation screening in this study did not apply Benjamini–Hochberg false discovery rate (FDR) correction for multiple testing. Correlations with |r| ≥ 0.5 and unadjusted *p* < 0.05 were retained for subsequent network construction and defined as key associations. Rare microbial taxa were defined as genera with average relative abundance below 1% across all samples.

## 3. Results and Discussion

### 3.1. Physical and Chemical Properties and Enzyme Activity

**Figure 1:**
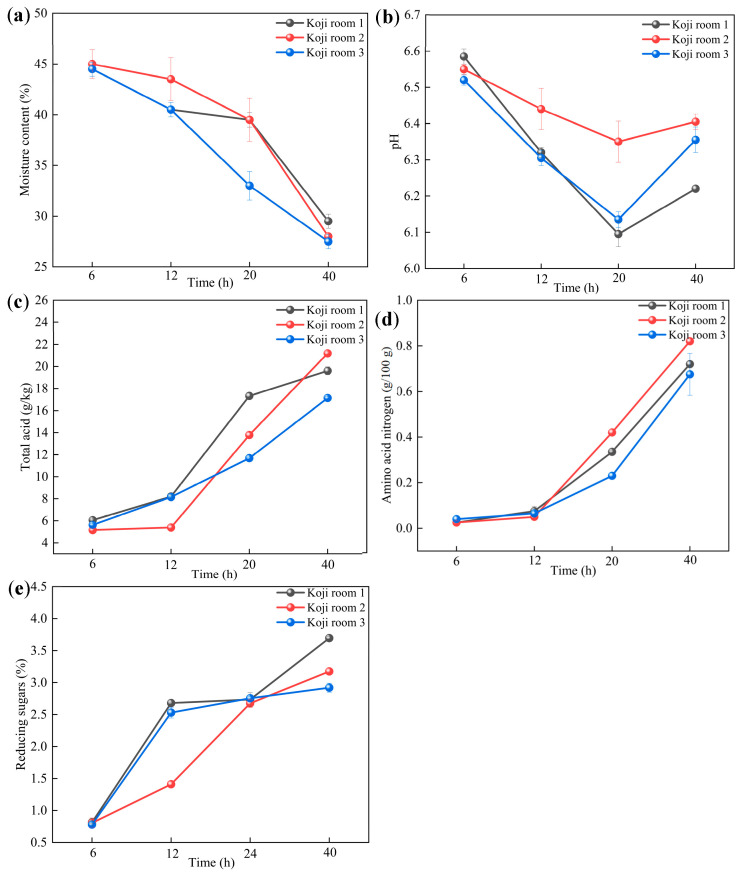
Physicochemical parameters of different koji rooms: (**a**) moisture content, (**b**) pH, (**c**) total acid, (**d**) amino acid nitrogen, (**e**) reducing sugars. All indicators were determined using standard analytical methods for fermented foods. Data are presented as mean ± standard deviation (SD) of three technical replicates.

As shown in Figure 1a, the moisture content in all three parallel koji rooms decreased significantly and continuously throughout fermentation. The declining rate increased markedly during the mid-to-late fermentation stages (20–40 h). Despite highly consistent overall trends, slight differences in the decreasing rate per unit time are observed across different koji rooms. This dynamic change is not simply a physical dehydration procedure but results from synergistic interactions of microbial metabolism, processing conditions, and physicochemical transformation within the koji matrix [23,24]. The intrinsic driving mechanism can be systematically interpreted from the coupling of microbial metabolism and processing parameters. As the fermentation period progresses, *Aspergillus oryzae* gradually enters its exponential growth phase and proliferates vigorously. Intense aerobic respiration of *Aspergillus oryzae* not only continuously consumes moisture in the system but also releases heat, causing the temperature of the koji substrate to rise continuously [25]. During the industrial production of koji, ventilation is used to maintain the temperature of the mixture within an appropriate range and prevent the koji from burning. Continuous ventilation lowers the temperature while accelerating the evaporation and loss of moisture from the material [26]. Meanwhile, hydrolytic enzymes, including amylase and protease, secreted by *Aspergillus oryzae* degrade macromolecular substrates in raw materials, destroy the compact granular structure of the matrix, and ultimately form a loose and porous koji structure. This structural change significantly increases the interfacial area between the koji and the ventilated air, thereby enhancing the diffusion and evaporation of moisture within the system [27]. The accompanying decline in water activity plays a dual role: it inhibits undesirable microorganisms, fostering a community where *Aspergillus oryzae* thrives and secretes essential enzymes, and it serves as a key benchmark for evaluating the quality and fitness of the finished koji [28,29]. The minor variations in moisture loss rates among the three parallel koji rooms primarily originate from slight deviations in process parameters during industrial-scale production. These deviations lead to subtle differences in microbial growth and metabolic intensity in each room, which are ultimately reflected in the observed variations in moisture change rates.

As shown in Figure 1b, the pH levels in all three koji rooms followed a pattern of initially decreasing and then rebounding. This process reflects changes in microbial metabolism and shifts in biochemical reaction equilibria [30,31]. During the early stages of koji production (0–20 h), *Aspergillus oryzae* proliferates rapidly and exhibits vigorous metabolic activity [32]. *Aspergillus oryzae* synthesizes organic acids such as citric acid and lactic acid through metabolic pathways including glycolysis and the tricarboxylic acid cycle; the continuous accumulation of these organic acids directly causes the pH of the system to decrease [8]. The observed pH increase starting at 20 h marked a metabolic transition. First, reduced sugar availability led to organic acid consumption [33]. Concurrently, extensive proteolysis by *Aspergillus oryzae* produced amino acids, whose subsequent microbial deamination released NH_3_ [34]. Combined acid consumption and ammonia production from deamination were associated with increased pH values. By 40 h, the pH values had stabilized, indicating that the growth and metabolism of *Aspergillus oryzae* had entered a steady state. Real-time pH monitoring enables effective evaluation of the koji production process, facilitates the early identification of process anomalies, and reduces the risk of microbial contamination. A low pH indicates excessive acidogenic bacteria growth, risking “sour koji”, while a high pH suggests ammonifying bacterial contamination, detrimental to flavor. Thus, real-time pH tracking is an essential diagnostic tool for process control and a cornerstone of consistent, high-quality koji production [35].

A steady increase in total acid content was observed across the three koji rooms (Figure 1c). The underlying driver of this trend is the microbial metabolism of *Aspergillus oryzae* and other koji microbiota, which synthesize organic acids—including citric, lactic, and acetic acids—through central metabolic pathways such as glycolysis and the tricarboxylic acid cycle [36]. The initial phase saw rapid organic acid production, while alkaline compound formation was limited [37]. This imbalance created an acid-dominant state that lowered the pH in parallel with increasing total acidity [38]. As the process progresses into the mid-to-late stages, total acidity continues to rise due to ongoing organic acid accumulation. However, *Aspergillus oryzae* simultaneously enters a peak protease-production phase. The enhanced proteolysis releases abundant amino acids, which undergo microbial deamination (e.g., from glutamine) to generate substantial alkaline ammonia (NH_3_). This ammonia neutralizes H^+^ ions released from organic acids, and this process is closely associated with the observed pH rebound during koji fermentation [34,39]. The organic acids produced during the koji process serve not only as physicochemical factors that regulate the environment of the system, but also maintain the competitive advantage of beneficial microorganisms, and inhibit the growth of unwanted bacteria. But the specific profile of organic acids ultimately formed (e.g., succinic acid and lactic acid) also works in conjunction with compounds like amino acids to form the flavor precursor framework of soy sauce fermentation [40,41]. Organic acids play dual roles: imparting a refreshing sourness and, through synergy with amino acids, creating a balanced taste foundation [42]. Furthermore, these free amino acids undergo conversion by microorganisms in subsequent fermentation, giving rise to the alcohols and esters that define soy sauce’s characteristic savory, rich, and layered flavor [43,44].

The amino acid nitrogen content in all three koji rooms showed an upward trend, with the rate of increase accelerating significantly during the middle to late stages of koji production (12–40 h) (Figure 1d). Amino acid nitrogen is a key quantitative indicator for assessing the total activity of proteolytic enzymes in the koji production system and the degree of protein degradation in the raw materials [45]. The amino acid nitrogen content not only directly reflects the accumulation of flavor-contributing amino acids such as glutamic acid and flavor precursors in the koji production, but also indirectly reflects the metabolic activity of microorganisms within the system [46,47,48]. This observed dynamic increase is largely explained by continuous proteolytic activity of microbial enzymes, notably those from *Aspergillus oryzae*, which contribute to the hydrolysis of substrate proteins [49]. During the early stages of koji production (0–12 h), *Aspergillus oryzae* secretes only limited amounts of proteases, resulting in the production of only small amounts of amino acids; consequently, amino acid nitrogen shows a slow upward trend. During the mid-to-late stages (12–40 h), the rapid proliferation of *Aspergillus oryzae* was accompanied by substantial secretion of endo- and exopeptidases. This enzymatic activity led to the extensive hydrolysis of proteins, generating large quantities of free amino acids and small peptides. Accordingly, the amino acid nitrogen concentration entered a phase of rapid accumulation, reaching 0.6–0.9 g/100 g by 40 h [50]. At the same time, the accumulation process of amino acid nitrogen provides direct experimental evidence for the U-shaped evolution mechanism of pH in the aforementioned koji production system. Even as total acidity continues to rise, the microbial deamination of amino acids—released from ongoing proteolysis—generates alkaline ammonia. This ammonia production is the key factor that reverses the pH decline, driving its rebound after the minimum point is reached [34].

The reducing sugar content in all three koji rooms showed a consistent upward trend overall, but there were significant differences in the accumulation dynamics of reducing sugars among the rooms (Figure 1e). The trends in reducing sugar levels in koji rooms 1 and 3 over the 0–20 h period closely mirrored their growth rates, whereas the accumulation of reducing sugar in koji room 2 exhibited a distinct two-phase pattern. The growth rate is relatively low, and the accumulation trend is gradual within the first 12 h; after 12 h, the growth rate accelerates significantly. At 40 h, the reducing sugar content in the three koji rooms was determined to be 3.69, 3.17, and 2.87 g/100 g, respectively. Reducing sugars serve as the primary carbon source for microbial growth and reproduction, and are also key flavor precursors in the Maillard reaction and yeast metabolism. Changes in reducing sugar content are the ultimate reflection of the dynamic balance between the production rate via hydrolysis of starch by microbial amylase and glucoamylase, and the consumption rate for microbial growth and metabolism [51,52]. In koji rooms 1 and 3, *Aspergillus oryzae* rapidly adapts to the environment, enters the active growth phase, and secretes large amounts of hydrolytic enzymes, efficiently breaking down the raw materials to produce reducing sugars. 0–12 h, the rate of reducing sugar consumption by *Aspergillus oryzae* was lower than the rate of production, resulting in a rapid accumulation of reducing sugars. However, the accumulation dynamics of reducing sugars in koji room 2 differed, primarily due to slight variations in microenvironmental parameters during the early stages of koji production. During industrial koji production, even slight variations in process parameters can affect the temperature and moisture content of the koji mixture, resulting in slow growth of *Aspergillus oryzae* in koji room 2 during the first 12 h of production. This leads to insufficient secretion of amylase and saccharifying enzymes, limited production of reducing sugars, and a gradual accumulation of these sugars. After 12 h, the metabolic and enzyme-producing capabilities of *Aspergillus oryzae* increase, leading to enhanced secretion of hydrolytic enzymes, accelerated starch hydrolysis, and a rapid rise in reducing sugar content. This indicates that precise control of process parameters during the early stages of mold production is crucial for ensuring synchronized growth of *Aspergillus oryzae*, stable enzyme production, and consistent raw material conversion efficiency across parallel koji rooms.

**Figure 2:**
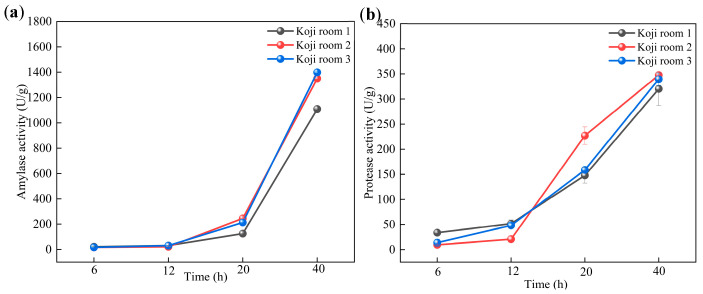
Changes in the activity of key enzymes in different koji rooms: (**a**) amylase activity in different samples; (**b**) protease activity in different samples. All indicators were determined using standard analytical methods for fermented foods. Data are presented as mean ± standard deviation (SD) of three technical replicates.

The amylase activity in all three koji rooms showed a steady increase as the fermentation process progressed (Figure 2a). The activity of the enzyme exhibited a two-phase pattern, with a slow increase in the early stage followed by a rapid rise in the later stage. During the first 20 h, amylase activity increased gradually, rising only from an initial level of 15–20 U/g to 100–250 U/g; from 20–40 h, it entered a period of rapid increase. At 40 h, amylase activity reached maximum values of 1108.56, 1349.75, and 1398.24 U/g in the three koji rooms, respectively. In the early stages, *Aspergillus oryzae* is in the adaptation phase, with a slow proliferation rate and the ability to synthesize only small amounts of amylase. In the middle and late stages, *Aspergillus oryzae* enters the growth phase, during which it proliferates rapidly while the rates of amylase synthesis and secretion accelerate, correlating with a rapid increase in enzyme activity [32,53]. Overall, the trends in amylase activity in koji rooms 2 and 3 were highly consistent, while the amylase activity in koji room 1 was slightly lower than that of the other two at each sampling time point. It is worth noting that the changes in reducing sugar content in the three rooms do not fully correspond to the trends in amylase activity. This is because the final content of reducing sugars in the system results from the dynamic balance between hydrolytic production and metabolic consumption. On the one hand, the formation of reducing sugars depends on the synergistic catalytic action of endo-amylase (which hydrolyzes starch internally) and exo-amylase (which hydrolyzes starch externally). On the other hand, the growth and reproduction of microorganisms such as *Aspergillus oryzae* consume reducing sugars; the combined effect of these two factors causes the trends in reducing sugar content and amylase activity to be out of sync [54,55,56].

Consistent with the changes in amylase activity, protease activity in the three koji rooms also showed a steady upward trend (Figure 2b). At the end of the 40 h koji production process, the values were 320.12, 347.53, and 339.60 U/g, respectively. The trends in protease activity in koji rooms 1 and 3 were similar, while koji room 2 showed slight variations. The dynamic changes in protease activity were consistent with the trends in amino acid nitrogen accumulation described earlier. This is consistent with the view that protein hydrolysis by proteases secreted by *Aspergillus oryzae* is a major contributor to the observed accumulation of free amino acids and the increase in amino acid nitrogen content in the fermentation system [57]. Amylase and protease constitute the core hydrolytic enzyme system in koji production. Their sustained synthesis and activity enable the degradation of raw material starch and proteins, thereby supplying essential substrates and flavor precursors for subsequent moromi fermentation and establishing the fundamental enzymatic basis for soy sauce production.

### 3.2. Analysis of Volatile Aromatic Compounds During Koji Production

#### 3.2.1. Composition of Volatile Flavor Compounds

**Figure 3:**
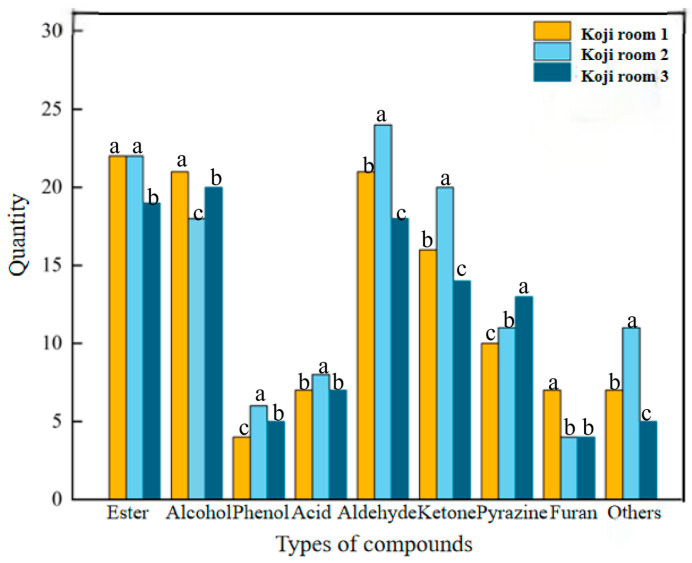
Summary of the types of odor compounds in different koji rooms. Volatile compounds were identified by GC×GC-O-MS and classified into 7 major chemical classes (alcohols, aldehydes, ketones, esters, acids, terpenes and others). Different lowercase letters indicate statistically significant differences in the total number of detected compound types or individual class counts between koji rooms (*p* < 0.05, one-way ANOVA with Tukey’s HSD test).

This study analyzed the volatile flavor compounds in three koji rooms, identifying the dynamic evolution patterns, common compositional characteristics, and distinctive features of flavor compounds across the different koji rooms. The results showed that there were significant differences in both the types and quantities of volatile odor-active substances detected in the three rooms (Figure 3). Koji room 1 identified 115 compounds, including 22 esters, 21 alcohols, 4 phenols, 7 acids, 21 aldehydes, 16 ketones, 10 pyrazines, 7 furans, and 7 other types of compounds. A total of 124 compounds were detected in koji room 2, including 22 esters, 18 alcohols, 6 phenols, 8 acids, 24 aldehydes, 20 ketones, 11 pyrazines, 4 furans, and 11 other compounds. A total of 105 compounds were detected in koji room 3, including 19 esters, 20 alcohols, 5 phenols, 7 acids, 18 aldehydes, 14 ketones, 13 pyrazines, 4 furans, and 5 other compounds. The divergence in flavor profiles among rooms is primarily attributable to the dynamic microbial succession and non-uniform microenvironment within the semi-open production system. A total of 77 volatile compounds were detected in the koji samples from the three koji rooms, including 14 esters, 15 alcohols, 4 phenols, 5 acids, 16 aldehydes, 10 ketones, 9 pyrazines, 1 furan, and 3 other types of compounds. Among these, 24 odor-active compounds were detected at all time points. They included phenethyl alcohol, phenethyl aldehyde, benzaldehyde, acetic acid, 4-vinylguaiacol, 1-octen-3-ol, etc. These substances form the flavor framework during the koji production stage of soy sauce production and establish the foundational flavor profile of the finished product. These results indicate that, although there are certain differences in the full spectrum of flavor compounds among the different parallel koji rooms, the composition of the core flavor compounds is highly consistent, ensuring the stability and uniformity of the final soy sauce product’s flavor characteristics from a flavor chemistry perspective.

Analysis of the quantitative results for various flavor compounds shows that alcohols (phenethyl alcohol, benzyl alcohol) and aldehydes (phenethyl aldehyde, benzaldehyde) are the two most abundant volatile components during the koji production process. The total concentrations of the two compounds reached average peak values of 2261.64 ng/g and 2787.33 ng/g, respectively, at 20 h into the koji production process. These compounds primarily originate from the metabolic conversion of amino acids by yeast via the Ehrlich pathway, as well as from byproducts of the Maillard reaction during the koji production process. Not only do they impart a distinctive, mellow aroma, floral and fruity notes, and rose-like scents to soy sauce, but they also serve as key precursors for the synthesis of ester-based flavor compounds during the subsequent fermentation stage of the soy sauce mash [58,59]. Among phenolic compounds, 4-vinylguaiacol (4-VG) has a smoky aroma and is a key contributor to the characteristic flavor of soy sauce [7]. Ester content remained low throughout (average peak: 383.05 ng/g), due primarily to limited yeast abundance during koji production, which constrained ester synthesis. In contrast, organic acids like acetic acid fulfill a dual role: they modulate microbial communities via pH and act as flavor precursors, collectively balancing the taste and complexity of soy sauce [60,61]. During the koji production process, the total content of esters, alcohols, aldehydes, ketones, and acids generally increases initially and then decreases, with most reaching their peak at 20 h into the process. These dynamic changes are coupled with the growth cycle of *Aspergillus oryzae*, changes in amylase and protease activity, and the rates of substrate degradation and consumption. In contrast, ketone compounds, due to their reactive chemical nature and susceptibility to microbial reduction, remain at relatively low levels throughout the process [6].

In summary, the formation and accumulation of volatile flavor compounds during the production of soy sauce starter are the result of the synergistic interaction of multiple processes, including microbial metabolic activity, enzymatic reactions (the degradation of macromolecular substrates mediated by proteases and amylases), and non-enzymatic chemical reactions (such as the Maillard reaction and Esterification) [62]. Among these, the 77 shared compounds—particularly the 24 that were persistently detected as flavor-active—collectively form the foundational flavor profile of koji. They serve as essential precursors for flavor development in subsequent fermentation and, more importantly, are critical to maintaining the relative consistency of final product quality in industrial-scale production.

#### 3.2.2. Analysis of Characteristic Flavor Compounds

To determine the key odorants in soy sauce koji, odor activity value (OAV) was calculated to identify compounds that define the overall aroma. OAV is defined as the ratio between volatile concentration and corresponding odor threshold and helps screen compounds with putative flavor relevance; an OAV ≥ 1 suggests potential sensory contribution rather than confirmed aroma activity [63]. The results showed that there were some differences in the number of key flavor-active compounds with OAV ≥ 1 across the different koji rooms: a total of 31 were detected in koji room 1 (Appendix A.2), 27 in koji room 2 (Appendix A.3), and 25 in koji room 3 (Appendix A.4). Thirteen volatile compounds were persistently identified across all koji rooms and time points, each with an OAV ≥ 1 throughout the koji production stage. These key odorants—including 1-octen-3-ol, ethyl acetate, 3-methylbutyraldehyde, hexanal, nonanal, decanal, phenethyl aldehyde, guaiacol, 4-VG, 2,3-butanedione, acetoin, and 2-pentylfuran—collectively form the core aroma profile of koji. They provide a critical and stable contribution to the foundational sensory characteristics of soy sauce. The formation pathways of these key flavor-active compounds are diverse; through the synergistic interaction of microbial metabolism and non-enzymatic chemical reactions, they collectively shape the complex and harmonious flavor profile of soy sauce. Alcohols such as 1-octen-3-ol are primarily generated via the Ehrlich pathway through amino acid degradation by yeasts, imparting a rich, characteristic alcoholic and fermented aroma to soy sauce. Aldehydes, including phenethyl aldehyde and 3-methylbutyraldehyde, mainly arise from the oxidative degradation of fatty acids and the Strecker degradation of amino acids. They contribute pleasant floral and fruity notes, constituting the core aromatic components of soy sauce [64,65]. The characteristic smoky aroma of 4-VG significantly enhances the depth and complexity of soy sauce. Meanwhile, esters, ketones, and furan compounds—generated primarily through enzymatic (e.g., esterification) and non-enzymatic (e.g., Maillard) reactions—further diversify and enrich the aromatic profile [66,67,68]. The ongoing accumulation of these key compounds during koji production provides the essential chemical foundation for their subsequent transformation and synergy in the moromi stage. This underscores the foundational role of koji production in shaping the final flavor profile, effectively tracing the origins of soy sauce flavor to this initial process.

### 3.3. Determination of Free Amino Acid Content During Koji Production

The sweetness of soy sauce is primarily derived from sweet-tasting amino acids (e.g., alanine (Ala), serine (Ser), and lysine (Lys)), reducing sugars (e.g., glucose, maltose, and sucrose), and some small-molecule peptides. Bitterness mainly originates from bitter-tasting amino acids (e.g., arginine (Arg), tyrosine (Tyr), phenylalanine (Phe), and isoleucine (Ile)), and certain peptides also contribute to bitterness [16]. In this study, the contents of 16 free amino acids during koji fermentation were determined and analyzed (Appendix A.5).

Overall, the 16 free amino acids in the three koji rooms exhibited similar changing trends. The total amino acid content increased significantly with fermentation time, but obvious differences in amino acid content were observed between different koji rooms at the same fermentation time point. Among them, arginine (Arg) exhibited a distinct trend from the other 15 amino acids. Its content fluctuated in all three koji rooms and reached the peak at 40 h of fermentation. At 12 h of fermentation, the content of most amino acids began to rise, and the proportion of bitter amino acids increased slightly. The period from 20 h to 40 h was the fastest growth phase for amino acid content, indicating that the late stage of koji making is the critical period for amino acid accumulation. At 40 h of fermentation, the total amino acid content was significantly higher than that in the previous three stages. At this time, the total amino acid content among the three koji rooms followed the order: Koji Room 2 > Koji Room 1 > Koji Room 3. This result was probably due to the more stable temperature and humidity and higher protease activity in Koji Room 2, which facilitated amino acid accumulation.

Among the three taste-active amino acid groups, umami amino acids (aspartic acid (Asp) and glutamic acid (Glu)) had the highest content, which is an important material basis for the umami characteristic of soy sauce. Compared with other amino acids, aspartic acid and glutamic acid showed a more significant increase, especially in the middle and late stages of fermentation, where their contents rose rapidly. In addition, although the content of bitter amino acids was lower than that of umami amino acids, it showed an overall steady increasing trend. The content of sweet-tasting amino acids showed a slight increase, and the content of sweet-tasting amino acids in Koji Room 3 was significantly lower than that in the other two koji rooms at all fermentation time points. This differential accumulation further indicates that the accumulation of different amino acids during koji fermentation is susceptible to environmental factors and microbial community structure.

### 3.4. Analysis of Microbial Diversity During Koji Production

#### 3.4.1. Microbial Alpha Diversity

As shown in Table 1, the Shannon diversity index of eukaryotic communities remained low, while the Simpson dominance index was consistently high throughout the entire koji production cycle. This indicates a low-diversity, high-dominance eukaryotic community structure across all koji rooms and time points, which is a direct outcome of the koji production process designed to select for a functional fungi consortium dominated by *Aspergillus oryzae*. Community dynamics analysis of the parallel koji rooms revealed that the Shannon and Simpson indices for koji room 2 remained notably stable throughout the fermentation cycle. This stability reflects a well-controlled community structure, which aligns with the primary goal of the koji process: selectively establishing a dominant *Aspergillus oryzae* population. In contrast, koji rooms 1 and 3 showed greater fluctuations in both indices, suggesting a stronger disruptive influence from environmental eukaryotic contaminants and less effective community control compared to koji room 2. By the end of koji production, the Shannon index dropped to minimal levels, and the Simpson index reached 1.00 in both rooms 2 and 3. This demonstrates the establishment of a highly homogeneous eukaryotic community dominated by the inoculated *Aspergillus oryzae*, thereby achieving the core technical objective of the koji stage [69]. Analysis of the Chao1 index (reflecting species richness) yielded conclusions consistent with the diversity indices. Except for a slightly higher value at the 6 h mark, koji room 2 consistently exhibited a lower Chao1 index than rooms 1 and 3 throughout fermentation, reaching its minimum at the endpoint. This pattern further reinforces the precise regulation favoring *Aspergillus oryzae* dominance in koji room 2.

Unlike the eukaryotic communities, prokaryotic microbiota in koji production are far more diverse and heterogeneous. Alpha diversity indices showed significant room-to-room differences and divergent temporal trends. This is a direct consequence of the semi-open system’s inherent lack of sterility, which facilitates the continuous introduction and establishment of environmental bacteria, thereby shaping a complex and variable bacterial consortium [70]. Given the significant ecological niche differentiation between prokaryotic and eukaryotic microorganisms in terms of physiological and metabolic characteristics, nutrient utilization strategies, and microenvironmental requirements, the eukaryotic dominant community—centered on *Aspergillus oryzae*—has not exerted a strong inhibitory effect on the diversity and species richness of the prokaryotic community, nor has it significantly shaped it [71]. Biomass measurements showed that eukaryotic microorganisms (overwhelmingly *Aspergillus oryzae*) were dominant, while prokaryotes occupied a subordinate position in terms of abundance. Consequently, their considerable diversity is manifested primarily as taxonomic richness, not as high population density or ecological dominance.

In summary, the dynamic succession of microbial communities during koji production fully reflects the core objective of industrial-scale production: the directed cultivation of *Aspergillus oryzae*. At the eukaryotic level, process control has enabled the targeted enrichment and dominance of the desired functional microorganisms. At the prokaryotic level, the semi-open production environment has allowed for the retention of high species diversity, resulting in a unique microbial community pattern characterized by “high enrichment of eukaryotic target functional microorganisms alongside a diverse and dynamic prokaryotic background community.” This community structure likely represents the core microbiological basis for the formation and accumulation of characteristic flavor precursors during the koji production stage of soy sauce production.

#### 3.4.2. Microbial β-Diversity During the Koji Production Process

**Figure 4:**
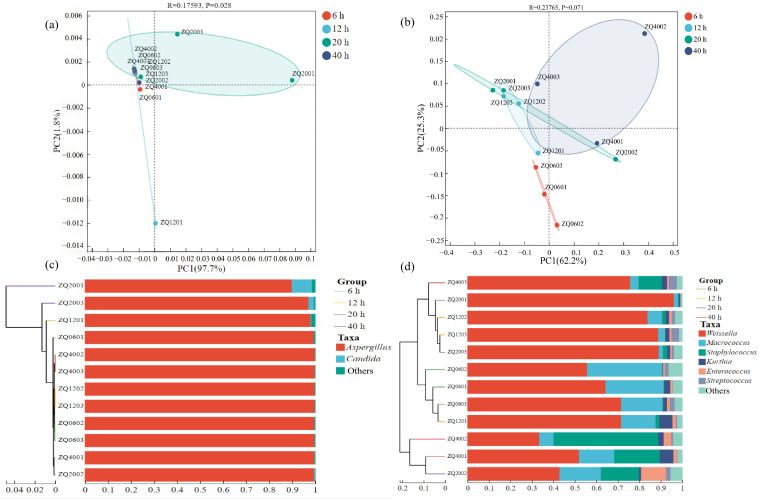
PCoA and hierarchical cluster analysis of bacteria and fungi during koji production. (**a**) Principal component analysis of bacterial composition during the koji production process, (**b**) principal component analysis of fungal composition during the koji production process, (**c**) hierarchical cluster analysis of bacterial composition during the koji production process, (**d**) hierarchical cluster analysis of fungal composition during the koji production process.

Based on principal coordinate analysis (PCoA) and hierarchical cluster analysis using the Bray–Curtis distance algorithm, this study systematically examined the spatiotemporal succession patterns of eukaryotic and prokaryotic microbial communities in samples collected from different koji rooms throughout the complete production cycle of soy sauce koji. The results show that the two types of microbial communities exhibited distinctly different patterns of dynamic succession during the koji production process (Figure 4). At the eukaryotic microbial level (Figure 4a), the community structures in different koji rooms were highly similar during the early stage of fermentation (6 h), confirming that the initial fermentation conditions were highly uniform across all parallel rooms. As the koji production process progresses, the eukaryotic communities’ structures in different koji rooms undergo temporary differentiation due to the inevitable microenvironmental heterogeneity resulting from temperature and humidity control, ventilation, and turning operations in each room. However, by the end of the fermentation process, the sample points from all koji rooms once again showed significant clustering, indicating that the community structure had returned to a high degree of uniformity. This dynamic succession pattern clearly indicates that, despite temporary microenvironmental fluctuations during koji production, all process operations—including artificial temperature control, ventilation, humidity regulation, and koji turning—are centered on promoting the growth of *Aspergillus oryzae*. This selective pressure ultimately drives the eukaryotic microbial communities from different koji rooms to converge by the end of the fermentation process, fully confirming that the process effectively achieves its core objective of establishing *Aspergillus oryzae* as the dominant microbial community [72,73]. Prokaryotic microbial communities (Figure 4b), however, exhibit a completely different pattern of succession. In the early stage (6 h), the prokaryotic microbial community structures in different rooms also exhibited a high degree of similarity. Although pure-culture inoculation is employed in koji production, the semi-open production environment introduces a small number of prokaryotic microorganisms from the surroundings [74]. As the koji process progressed, the differences in the original microbial community structures among the different koji rooms gradually increased, with no convergence observed by the end of the process.

The results of hierarchical cluster analysis further validated and refined the patterns of microbial community succession revealed by PCoA. Regarding the eukaryotic microbial communities, with the exception of a few samples—such as ZQ2001, ZQ2003, and ZQ1201—that exhibited some variation, the vast majority of samples clustered closely together in the phylogenetic tree. In contrast, hierarchical clustering of prokaryotic microbial samples revealed significant differentiation, with the samples clearly separating into multiple distinct clusters. Among them, ZQ4003, ZQ2001, ZQ1202, ZQ1203, and ZQ2003 formed one cluster; ZQ0602, ZQ0601, ZQ0603, and ZQ1201 formed another cluster. While ZQ4002, ZQ4001, and ZQ2002 formed an independent cluster. This clustering pattern is fully consistent with the results of the PCoA analysis. Together, these findings confirm that although the composition of prokaryotic microorganisms in each fermentation koji room was highly similar at the beginning of the process, their successional trajectories diverged markedly as fermentation progressed. Under the unified control exerted by the growth of *Aspergillus oryzae*, the prokaryotic communities in different rooms evolved along distinct pathways due to the combined effects of microenvironmental heterogeneity and interspecies interactions, with structural differences continuing to widen over time.

Furthermore, the results of PCoA and hierarchical cluster analysis jointly indicate that the differences in microbial communities among industrial parallel koji rooms are primarily concentrated at the prokaryotic level. As the key component in koji production, *Aspergillus oryzae* achieves a high degree of consistency and uniformity by the end of the process. Perspective of standardizing the koji production process and ensuring product quality consistency, optimizing environmental control measures—such as air sterilization in koji rooms and surface disinfection of production equipment—can effectively reduce the initial load of environmental prokaryotic microorganisms. This, in turn, minimizes random interference from non-target microorganisms during koji production, further enhancing the consistency of microbial communities across parallel koji rooms and ensuring the uniformity and stability of the final koji quality.

#### 3.4.3. Analysis of Microbial Community Structure During Koji Production

**Figure 5:**
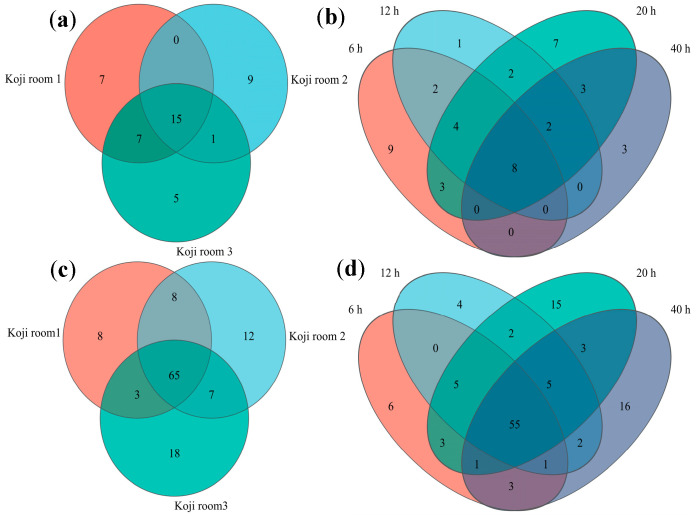
Venn diagram of fungi and bacteria during the production of soy sauce koji. (**a**) Venn diagram of fungi from different koji rooms, (**b**) Venn diagram of fungi from different times, (**c**) Venn diagram of bacteria from different koji rooms, (**d**) Venn diagram of bacteria from different times.

Venn diagram analysis based on species composition (Figure 5) further revealed the core differences and intrinsic characteristics of eukaryotic and prokaryotic microbial community structures during the koji production process, as examined from the perspectives of species composition and dynamic succession. At the eukaryotic microorganism level (Figure 5a), the three parallel fermentation rooms exhibited comparably low total observed species richness. Specifically, 29, 25 and 28 eukaryotic species were detected in Rooms 1, 2 and 3, respectively. In total, 44 eukaryotic species were recovered from all tested samples, and species shared by all three rooms accounted for 34.1% of the overall detected species pool. These results indicate that the koji production process, which focuses on the targeted cultivation of *Aspergillus oryzae*, has shaped a eukaryotic community framework. This framework is characterized by a relatively simple structure and distinct shared features, and is consistent with the findings from the Alpha diversity and PCoA analyses described earlier. Although overall fungal community structures were highly similar among the different koji rooms, each room contained a small set of unique endemic species. Room 2 has the highest number of endemic species (9), and species richness fluctuates dynamically across different stages of the fermentation process (Figure 5b). These observations tentatively suggest that, even within the main process designed for the directed cultivation of *Aspergillus oryzae*, subtle microenvironment discrepancies between individual koji rooms may be associated with localized shifts in fungal community composition.

Prokaryotic microbial communities, in contrast, exhibit significantly greater complexity and dynamic variability (Figure 5c). The total detected richness of prokaryotic taxa in the three koji rooms was significantly higher than that of eukaryotes. A total of 84, 92, and 93 prokaryotic species were detected in koji rooms 1, 2, and 3, respectively. Across all samples, a cumulative total of 121 prokaryotic species were detected, of which 65 were shared among the three rooms, accounting for 53.7% of the total species count. These shared species constitute a core bacterial species pool with a high degree of sharing within the koji production system. At the same time, the number of endemic prokaryotic species was significantly higher in each koji room, with koji room 3 alone hosting as many as 18 endemic species, suggesting that the prokaryotic communities possess greater potential for differentiation. From the perspective of species dynamics throughout the entire koji production cycle (Figure 5d), the total number of prokaryotic species showed a steady increase as the process progressed, rising from 74 species at the 6 h mark in the early stage to 86 species at the 40 h mark at the end of the process. Furthermore, the number of unique species that emerged during the middle and late stages (20–40 h) was 15 and 16, respectively, which was significantly higher than that in the early stage. This pattern is fully consistent with the results of the Alpha diversity analysis discussed earlier.

In summary, the analysis of species composition further confirms that the koji production process exerts differential regulatory effects on the two types of microbial communities. Specifically, the manufacturing process appears to constrain the overall structure of the eukaryotic communities and favors compositional convergence of eukaryotic assemblages among different koji rooms. By contrast, directional shaping of prokaryotic communities by this process appears relatively limited, a trend that coincides with community profiles featuring elevated taxonomic richness, stronger temporal variability, and more distinct inter-room differentiation [75]. These inherent disparities in community composition and succession between eukaryotic and prokaryotic assemblages may serve as a useful reference for exploring associations between artificial targeted regulation and spontaneous microbial succession in industrial koji production.

**Figure 6:**
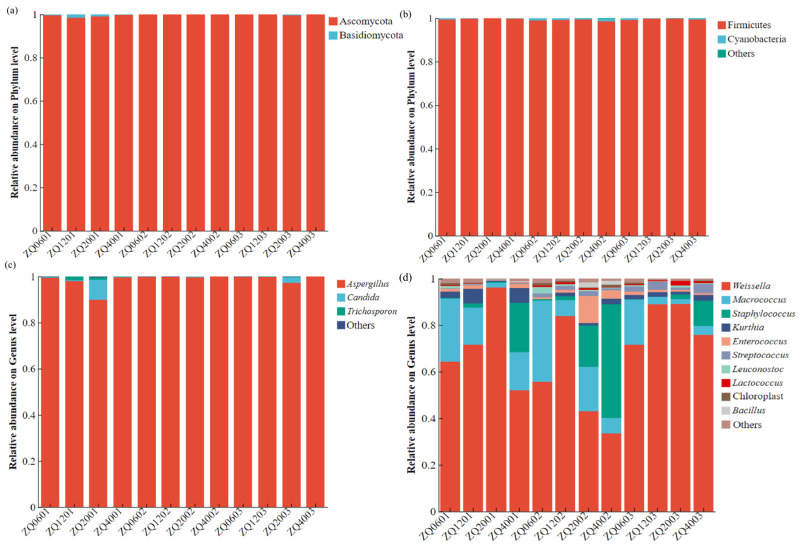
Changes in bacterial communities and fungal structures at the phylum and genus levels during the koji production process. (**a**) Phylum-level analysis of fungi during the koji production process, (**b**) phylum-level analysis of bacteria during the koji production process, (**c**) genus-level analysis of fungi during the koji production process, (**d**) genus-level analysis of bacteria during the koji production process.

Analysis of eukaryotic and prokaryotic communities throughout soy sauce koji production, with a relative-abundance cutoff of relative abundance greater than 1%, yielded the following findings (Figure 6). The two types of microbial communities exhibit strikingly different characteristics in terms of their structural composition and dynamic changes.

For eukaryotic communities, Ascomycota and Basidiomycota are the only core phyla. Among these, Ascomycota accounted for 98.44% to 99.95% of the relative abundance across all samples, demonstrating absolute dominance. This finding aligns with the core objective of industrial koji production, which is to selectively enrich filamentous fungi and cultivate dominant *Aspergillus oryzae* populations [69]. Basidiomycota were detected only in some samples, primarily in the 12 h and 20 h samples from koji room 1. They exhibited significant spatiotemporal specificity, which is presumed to be closely related to differences in microenvironmental regulation among the various rooms. Genus-level analysis shows that the dominant fungi in the koji production system are primarily concentrated in the genera *Aspergillus oryzae*, *Candida* and *Saccharomyces*. Among these, *Aspergillus oryzae* was the dominant genus, while *Candida* exhibited relatively high abundance at certain stages of the koji production process (particularly in the 20 h sample from koji room 1). Earlier reports have linked *Candida* spp. to salt-tolerant traits and putative flavor improvement during fermentation. Members of this genus co-vary with the accumulation of lactic, succinic and citric acids, which are associated with mild acidic notes and balanced sensory profiles of koji-derived soy sauce. Certain yeast strains, such as *Candida etchellsii*, synthesize characteristic flavor compounds like 4-ethylguaiacol and 4-hydroxy-2-ethyl-5-methyl-3-furanone (HEMF), which contribute smoky and caramel-like notes. Furthermore, exogenous supplementation of this genus in the mid-to-late fermentation stage has been reported to correlate with elevated contents of major volatile flavor compounds and umami-related indices (e.g., amino acid nitrogen) in soy sauce. Together with yeasts such as *Zygosaccharomyces rouxii*, taxa from this genus are suggested to constitute part of the core functional consortium, whose metabolic interactions may be associated with the formation of abundant and sophisticated soy sauce aroma characteristics [76,77,78]. *Saccharomycopsis* was almost exclusively detected in samples from Koji Room 1 at 12 h, and showed extremely low abundance or was not found in the other samples. The findings of this study closely align with those reported by Han et al. [19] regarding the microbial ecology of soy sauce koji. Together, they confirm the general ecological pattern in *Aspergillus oryzae*-centered systems. Eukaryotic communities are dominated by the target genus and exhibit relatively low complexity, alongside a dynamic minority of conditionally symbiotic or contaminating species.

For prokaryotic communities, only two phyla—Firmicutes and Cyanobacteria—were detected with relative abundances exceeding 1%. Firmicutes were the dominant phylum at all stages of koji production, with a relative abundance consistently above 98%. Cyanobacteria mainly appeared in the early stage of koji production and were continuously detected only at specific time points in individual koji rooms, with an overall extremely low abundance. At the genus level, 11 bacterial taxa presented relative abundance above the 1% screening threshold. *Weissella* appeared to prevail across all samples, with its relative abundance following an observed unimodal trend of rising first and declining thereafter over the koji fermentation. As a prevalent lactic acid bacterial taxon within the koji system, *Weissella* secretes proteases and amylases to degrade soy protein and starch, contributing to the accumulation of amino acid nitrogen. Simultaneously, taxa affiliated to *Weissella* have been reported to catabolize sugars for the synthesis of organic acids, including lactic and acetic acid, which may correlate with shifts in environmental pH and are potentially linked to suppressed proliferation of unwanted microbial taxa [79]. In addition, *Weissella* can synthesize extracellular polysaccharides such as β-glucans, which are potentially associated with improvements in color and viscosity of soy sauce. *Weissella* are reported to interact with *Aspergillus oryzae*, and such interactions may be associated with elevated hydrolytic enzyme activity and improved protein utilization of raw materials of raw material proteins. Meanwhile, it metabolizes to produce volatile flavor compounds such as alcohols and esters, imparting fruity and estery aromas to the soy sauce. Certain strains can synthesize bacteriocins to inhibit spoilage bacteria and degrade biogenic amines, making *Weissella* a core group of functional microorganisms essential for flavor development, quality enhancement, and product safety control during the koji production process [80]. The average relative abundance of *Macrococcus* declined from 34.8% to 20.3%. Nevertheless, likely due to shifts in its physiological state and environmental conditions, it was progressively excluded during koji production and yielded minimal direct contribution to the final flavor of soy sauce.

*Staphylococcus* abundance was initially low but increased significantly by the end of koji production. As a halotolerant functional group, *Staphylococcus* has been reported to catabolize available substrates and form organic acids (e.g., lactic, acetic, succinic), and these metabolites are potentially associated with desirable sourness and mellow sweetness in soy sauce. Acidification driven by such metabolic activity may correlate with suppressed proliferation of spoilage taxa and elevated ester formation. Nevertheless, modulation of salt content and temperature is commonly applied to restrain excessive multiplication of these organisms, as unchecked overgrowth has been linked to undesirable turbidity and off-flavor formation in finished products [81,82]. The genus *Kurthia* was detected only during the early stages of koji production, and its abundance declined rapidly as the process progressed. It is speculated that this genus may be involved in the initial decomposition of raw materials and community formation during the early stages of koji production. However, current research has not yet clarified its specific metabolic functions, and its contribution to the primary flavor profile of soy sauce is limited. *Enterococcus* maintained a high abundance in samples from all koji rooms, with koji room 2 showing the most significant levels. Members of this genus have been reported to interact with *Aspergillus oryzae*, and such interactions may be associated with elevated protease activity in koji, accelerated breakdown of raw-material macromolecules, and higher contents of nutritional markers, including amino acid nitrogen, total nitrogen, reducing sugars, polyphenols, and flavonoids. Salt- and acid-tolerant members of this genus have been reported to produce organic acids, and this metabolic trait may be associated with pH fluctuation and the suppressed proliferation of undesirable microbial taxa. Furthermore, safety evaluations have confirmed that it is non-pathogenic, making *Enterococcus* one of the core functional microbial groups in the koji production and subsequent fermentation stages of soy sauce production [83]. *Streptococcus* was more abundant in rooms 2 and 3 (notably koji room 3) than in koji room 1. This genus has been reported to catabolize sugars into organic acids, which may correlate with pH variation and the suppressed proliferation of acid-sensitive undesirable taxa. Such organic acids are presumed to act as ester precursors that could interact with alcohols to generate aroma compounds, including ethyl acetate [84]. Additionally, its proteolytic activity releases free amino acids that enhance umami. However, its growth must be carefully managed to prevent excessive acidification from hindering the core metabolism of *Aspergillus oryzae* and yeasts [85,86]. Although the genus *Leuconostoc* is not particularly abundant in the system, it remains consistently detectable until the end of the koji production process. *Leuconostoc* has been reported to yield lactic acid, ethanol and CO_2_ via heterofermentative lactic acid fermentation. Organic lactic acid originating from the metabolism of these taxa has been reported to interact with yeast-derived ethanol, and such interactions are potentially associated with the formation of lactate esters as well as the mild acidic and esteric sensory characteristics of soy sauce. Additionally, the metabolic activity of these taxa may correlate with reduced mash pH, and such pH shifts are presumably linked to the favorable progression of soy sauce moromi maturation. Accordingly, taxa affiliated with *Leuconostoc* are considered a promising functional resource for prospective flavor modulation of soy sauce [87,88]. *Lactococcus* showed distinct dynamics across rooms: abundant early in koji room 1 but largely eliminated later; persistently present in rooms 2 and 3, remaining detectable in room 3 at the endpoint. As commonly reported lactic acid bacterial taxa, members of this genus are suggested to exert diverse functions: (1) rapid acidification via homolactic fermentation inhibits pathogens (e.g., *E. coli*); (2) its proteolytic system boosts amino acid nitrogen; (3) its metabolic lactic acid is esterified to flavor compounds; (4) some strains produce bacteriocins like nisin; (5) its salt tolerance makes it a functional mainstay in the high-salt koji system [89,90]. The genus *Bacillus* is primarily concentrated in koji room 2 and is one of the core functional microbial groups in soy sauce fermentation. Members of this genus have been reported to produce proteases and amylases, and the occurrence of these enzymes may be associated with enhanced hydrolysis of macromolecules and higher concentrations of amino acid nitrogen and reducing sugars. It also metabolizes to produce flavor compounds such as pyrazines, working synergistically with yeast to enhance the flavor of soy sauce. However, certain strains (e.g., *Bacillus subtilis*) may produce undesirable flavors such as butyric acid. Furthermore, because *Bacillus* spores are heat-resistant and difficult to completely inactivate, it is necessary to screen for specific strains and strictly control fermentation conditions to mitigate these risks [91].

In summary, the eukaryotic and prokaryotic microbial communities in the koji production system exhibit distinctly different structural characteristics and successional patterns. The eukaryotic communities are highly simplified in structure, with the target functional genus *Aspergillus* dominating overwhelmingly, thereby fully achieving the core objective of artificial, directed regulation. In contrast, the prokaryotic microbial community is dominated by the phylum Firmicutes, with multiple functional genera comprising a complex and dynamic bacterial ecosystem characterized by significant spatial differentiation. *Weissella* is one core functional taxon participating in major biochemical reactions of koji production. A varied succession of other genera may partly account for slight discrepancies in physicochemical parameters and flavor precursor accumulation among different koji rooms.

### 3.5. Correlation Analysis

#### 3.5.1. Analysis of the Relationship Between Microorganisms and Key Flavor Compounds

**Figure 7:**
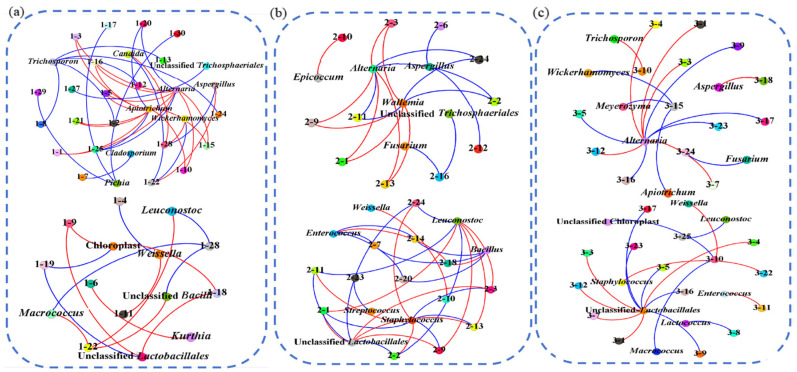
Correlation between characteristic flavor compounds and bacterial and fungal communities in different koji rooms. (**a**) Network diagram of dominant microorganisms and key flavor compounds in koji room 1, (**b**) network diagram of dominant microorganisms and key flavor compounds in koji room 2, (**c**) network diagram of dominant microorganisms and key flavor compounds in koji room 3. Only statistically significant correlations (|r| > 0.5, *p* < 0.05) are displayed in the networks. Red lines denote negative correlations, while blue lines denote positive correlations.

To further elucidate the intrinsic relationship between microbial community structure and the formation of volatile flavor compounds during the production of soy sauce koji, this study selected the top 10 eukaryotic and prokaryotic microbial taxa ranked by genus-level relative abundance and performed a Spearman rank correlation analysis with key odor-active volatile compounds [92]. The results indicate that the microbial–flavor compound correlation networks in the three parallel koji rooms exhibit significant heterogeneity, which is closely related to the differentiated succession patterns of prokaryotic and eukaryotic microbial communities across the different rooms (Figure 7). During industrial koji fermentation, even slight fluctuations in cultivation conditions can significantly affect the growth, metabolism, and interspecies interaction patterns of multi-microbial communities, thereby driving the differential evolution of microbial community structure and function. This also represents one of the core reasons why precise control is difficult to achieve in multi-microbial mixed fermentation processes within semi-open systems [93].

In koji room 1, *Aspergillus*, the genus with the highest relative abundance, showed a significant positive correlation only with 2-pentylfuran. The second most abundant genus, *Candida*, showed a significant positive correlation only with the flavor compound 2,3-butanone. In contrast, the less abundant genera *Saccharomycopsis*, *Apiotrichum* and *Alternaria* exhibited significant correlations with a greater number of key flavor-active compounds. Among prokaryotic microbial groups, the overall associations between microorganisms and flavor compounds were relatively weak. *Weissella*, the genus with the highest relative abundance, was significantly positively correlated only with maltol and significantly negatively correlated with hexanal. In contrast, the less abundant groups Unclassified *Lactobacillales* and Unclassified Chloroplast were each significantly associated with three flavor compounds. These results suggest that the high-abundance microbial communities, which dominate the biomass in the system, may not directly participate in the synthesis of characteristic flavor compounds. Rather, their core function appears to be the breakdown of macromolecular raw materials to provide usable precursor substrates for the flavor-synthesizing metabolism of low-abundance microorganisms. The correlation network for koji room 2 exhibits characteristics similar to those of koji room 1, albeit with significant differences. Among eukaryotic microbial taxa, the most abundant genus, *Aspergillus*, showed significant correlations only with the flavor compounds ethyl acetate and decanal. In contrast, the less abundant genera *Alternaria* and *Wallemia* exhibited significant correlations with multiple key flavor compounds. Among prokaryotic microbial taxa, *Staphylococcus*, *Leuconostoc*, and Unclassified *Lactobacillales* showed significant correlations with most flavor-active compounds, whereas *Weissella*, which had the highest relative abundance, showed a significant negative correlation only with the flavor compound hexanal. The correlation network for koji room 3 further underscores the heterogeneity in association patterns across parallel rooms. Among eukaryotic taxa, only *Alternaria*—despite its low abundance—showed significant associations with multiple flavor compounds. Among prokaryotic taxa, only Unclassified *Lactobacillales* exhibited significant correlations with several flavor-active compounds.

Taken together, these results reveal a key principle in mixed-culture fermentation systems: the microbial groups that make a direct and significant contribution to the formation of soy sauce’s characteristic flavor are not necessarily those that dominate the system in terms of biomass. High-abundance core functional microbial genera (e.g., *Aspergillus* and *Weissella*) tend to occupy a core ecological niche characterized by the primary catabolic breakdown of macromolecules such as starch and protein, thereby providing utilizable nutrient substrates and precursors for the growth, metabolism, and flavor synthesis of the entire microbial community. Meanwhile, a large number of rare microbial taxa present at low concentrations can utilize these precursor substrates to synthesize key flavor-active compounds through characteristic secondary metabolic pathways, which play a decisive role in shaping the aroma profile of soy sauce [94]. Correlation analysis can only reflect statistical associations between microorganisms and flavor compounds; the underlying regulatory mechanisms between the two are far more complex than these apparent associations, and further clarification is needed through subsequent functional validation using pure cultures [95,96]. The marked heterogeneity in correlation networks among parallel koji rooms originates in subtle variations in process parameters and environmental conditions. These variations alter interspecies microbial interactions, which in turn lead to the divergent correlation patterns observed between specific microorganisms and flavor compounds. From a microbial ecology perspective, these findings reveal the mechanisms underlying the distinctive and difficult-to-replicate flavor profiles of naturally fermented foods involving multiple strains. They also provide important scientific evidence for stabilizing the quality of industrially produced soy sauce through flavor-targeted regulation and finished product blending.

#### 3.5.2. Analysis of the Correlation Between Microbiological and Physicochemical Parameters

**Figure 8:**
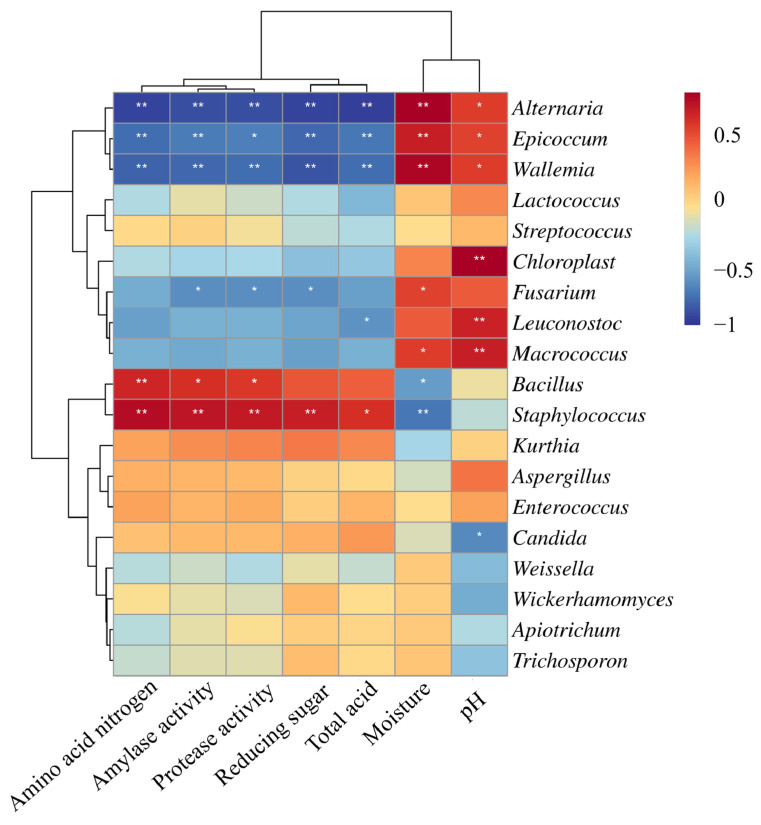
Correlation between physicochemical parameters and bacterial and fungal communities. *p*-values less than 0.05 are marked with *; 0.01 < *p* ≤ 0.05 with **. Only statistically significant correlations (|r| > 0.5, *p* < 0.05) are displayed in the networks.

To further elucidate the intrinsic relationship between microbial community succession and changes in key physicochemical indicators during soy sauce koji production, we selected the top 10 dominant microbial genera by relative abundance and performed Spearman’s rank correlation analysis with key physicochemical indicators measured throughout the entire production cycle, including moisture content, pH, total acidity, amino acid nitrogen content, reducing sugar content, amylase activity, and protease activity. Based on the analysis results, a correlation heatmap was generated (Figure 8). In the heatmap, the intensity of the colors represents the strength of the correlation, with warm colors indicating a positive correlation and cool colors indicating a negative correlation. Results indicated that pH and moisture content exerted similar trends in their associations with microbial community structure. Both parameters showed consistent positive correlations with a variety of microbial taxa. *Leuconostoc* and *Macrococcus* exhibited a highly significant positive correlation with pH (r = 0.89 and 0.76, respectively; |*p*| < 0.01), indicating that their relative abundances decreased significantly as the system pH declined. This trend can be associated with organic acid production (e.g., lactic acid) by *Leuconostoc* via heterofermentative pathways. Heterofermentative lactic acid metabolism of *Leuconostoc* is likely linked to pH shifts across fermentation [97]. At the same time, the optimal growth pH range for this bacterial genus is 6.5–7.0, making it better suited to a slightly acidic growth environment; if the system pH is too low, it will disrupt the integrity of the cell structure, thereby significantly inhibiting the bacteria’s normal growth and metabolism. *Alternaria* and *Wallemia* showed a highly significant positive correlation with moisture content (r = 0.88 and 0.87; |*p*|< 0.001), indicating that the growth and proliferation of these two fungal genera are highly dependent on the high-moisture environment within the koji system. As moisture content continuously decreased during the process, their abundances declined significantly. In addition, *Candida* showed a significant negative correlation with pH (r = −0.66, |*p*|< 0.05). As the pH increased during the later stages of koji production, the relative abundance of this genus decreased significantly, indicating that this acidophilic genus is better adapted to the weakly acidic microenvironment of the early stages and that its growth and proliferation are highly dependent on acidic conditions. The levels of amino acid nitrogen, reducing sugars, amylase activity, and protease activity exhibited significantly strong positive correlations with *Staphylococcus* and *Bacillus*, and significantly strong negative correlations with *Alternaria*. These results are highly consistent with the findings of Zhang et al. [98]. Both *Staphylococcus* and *Bacillus* secrete highly active extracellular proteases that efficiently hydrolyze macromolecular proteins (e.g., soy protein) in the feedstock, thereby promoting the continuous accumulation of amino acid nitrogen in the system. Additionally, *Bacillus* species produce various amylases that hydrolyze starch-based polysaccharides to generate reducing sugars, thereby increasing reducing sugar content. This pattern is consistent with previous reports on the functional characteristics of microorganisms in soy sauce koji production systems [99,100,101].

## 4. Conclusions

This study systematically analyzed the synergistic changes in physicochemical properties, volatile flavor compounds and microbial communities during parallel koji production for industrial soy sauce. It also revealed the core mechanisms responsible for the overall consistency and minor quality differences among products from parallel batches. Research has confirmed that the core physicochemical indicators and changes in hydrolytic enzyme activity in the three parallel koji rooms show a high degree of consistency with the structure of the eukaryotic functional microbial community. Through targeted artificial regulation, the researchers successfully achieved the preferential enrichment of the target functional fungus, *Aspergillus oryzae*. Large amounts of flavor precursors and hydrolytic enzymes were accumulated in the base mash during fermentation, which provided abundant substances and enzymes for the further fermentation of the sauce mash. This work further clarifies that the U-shaped pH profile (an initial decline followed by a subsequent rise) during koji fermentation is closely associated with alkaline metabolites generated from protein degradation and deamination, which neutralize organic acids. These findings raise questions regarding the conventional assumption that pH dynamics alone can accurately estimate total organic acid concentrations. Koji production is a critical foundational stage for the formation of soy sauce flavor. Although the full spectrum of volatile flavor compounds varies somewhat across parallel koji rooms, the 77 common compounds and 13 consistently detected high-OAV key flavor compounds form the core flavor framework of soy sauce koji, ensuring the stability and uniformity of the final product’s flavor characteristics from a chemical standpoint. In the koji production system, eukaryotic and prokaryotic microbial communities exhibit distinctly different succession patterns. Under targeted process control, the structure of eukaryotic microbial communities becomes highly simplified, and the community structure at the end of the koji production process shows a high degree of convergence, thereby fully achieving the core process objectives of koji production. In contrast, due to the lack of targeted control, prokaryotic microbial communities exhibit greater species diversity and more random succession patterns; this is the primary microbiological factor underlying the subtle fluctuations in physicochemical parameters and the accumulation of flavor compounds between parallel koji rooms. In addition, this study clarified the functional division of labor among microorganisms in the koji production and mixed-culture fermentation system. High-abundance core microbial taxa are closely associated with the basic hydrolytic transformation of raw-material macromolecules, which supplies intermediate substrates and biosynthetic precursors supporting microbial proliferation and flavor formation. By contrast, low-abundance rare taxa show tighter covariation with the accumulation of predominant flavor-active constituents. Among these, *Staphylococcus* and *Bacillus* are important functional commensal genera in the koji production process. These findings provide scientific support for standardizing and optimizing industrial soy sauce koji production, as well as for refining environmental control strategies. In the future, selectively regulating the structure of commensal bacterial communities could further enhance the consistency of quality and flavor characteristics in soy sauce products.

Several limitations should be addressed. First, correlation networks were constructed without FDR correction, so associations between microbes, physicochemical parameters and volatiles are exploratory only; the putative functional divergence of abundant and rare taxa awaits verification via pure culture or multi-omics. Second, candidate odorants were screened by OAV using literature thresholds from mismatched matrices, and no sensory recombination/omission trials were performed to validate their actual aroma contribution. Third, semi-open industrial conditions caused uncontrollable microenvironmental interference, limiting precise quantification of environmental effects on microbiota. Finally, our interpretation of U-shaped pH variation relies solely on correlative data, failing to quantify the respective contributions of alkali generation and organic acid metabolism; hence, we only supplement existing empirical inference rather than overturn traditional approaches.

## Figures and Tables

**Table 1:** Alpha diversity index for soy sauce koji.

Sample		Bacteria				Fungi			
		Shannon	Simpson	Chao	Coverage	Shannon	Simpson	Chao	Coverage
6 h	Koji room 1	1.13	0.47	49.86	1.00	0.04	0.99	15.33	1.00
	Koji room 2	1.19	0.42	57.43	1.00	0.03	0.99	18.75	1.00
	Koji room 3	1.08	0.54	72.67	1.00	0.02	1.00	9.00	1.00
12 h	Koji room 1	1.12	0.53	58.50	1.00	0.11	0.96	14.33	1.00
	Koji room 2	0.84	0.70	66.50	1.00	0.02	1.00	12.00	1.00
	Koji room 3	0.61	0.78	61.17	1.00	0.02	1.00	14.33	1.00
20 h	Koji room 1	0.27	0.92	44.00	1.00	0.50	0.81	21.33	1.00
	Koji room 2	1.77	0.25	70.63	1.00	0.03	0.99	13.00	1.00
	Koji room 3	0.72	0.78	81.33	1.00	0.18	0.94	20.00	1.00
40 h	Koji room 1	1.58	0.32	100.63	1.00	0.11	0.96	14.00	1.00
	Koji room 2	1.61	0.32	73.43	1.00	0.002	1.00	5.00	1.00
	Koji room 3	1.14	0.57	58.80	1.00	0.006	1.00	9.00	1.00

**Figure A1:**
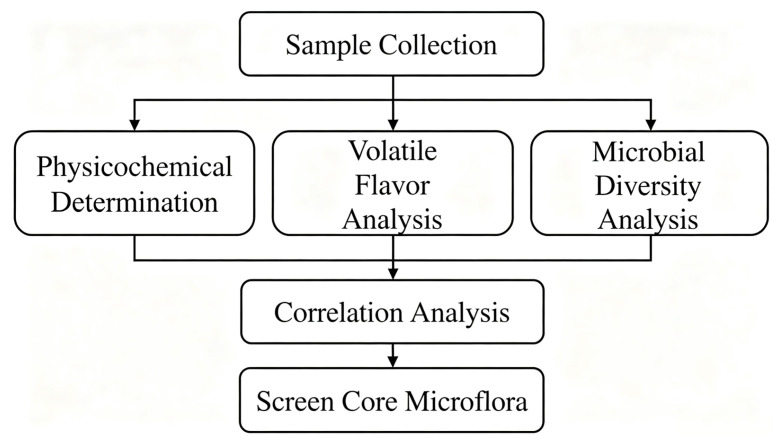
Schematic flowchart of the experimental technical route.

## Data Availability

The raw sequencing data from this study are available in the NCBI SRA database under BioProject accession numbers PRJNA1477102 and PRJNA1477124. The original contributions presented in this study are included in the article. Further inquiries can be directed to the corresponding authors.

## Appendix A

### Appendix A.2

**Table A1:** Key flavor compounds in koji room 1.

Number	Odor Characteristics	Compound	Testing Methods	ZQ0601	ZQ1201	ZQ2001	ZQ4001	Threshold (μg/L)
1-1	fruity	3-Methyl-1-butanol	MS/RI/O/ STD	0	0	2	3	220
1-2	green, fruity	1-Hexanol	MS/RI/O	1	2	1	0	5.6
1-3	mushroom	1-octen-3-ol	MS/RI/O/ STD	20	34	60	129	1.5
1-4	popcorn	Maltol	MS/RI/O/ STD	0	0	1	0	1240
1-5	sulfurous onion	3-Methylthiopropanol	MS/RI/O	0	0	3	3	123.23
1-6	fruity sweet	Acetic ether	MS/RI/O/ STD	8	8	9	4	5
1-7	-	2-Butyl acrylate	MS/RI	3	2	0	0	2
1-8	-	2-Ethylhexyl acrylate	MS/RI	3	5	4	2	5
1-9	fruity, creamy	Octadecyl ethyl ester	MS/RI/O	0	0	1	0	200
1-10	fruity	3-Methylbutyraldehyde	MS/RI/O	15	17	457	679	1.1
1-11	woody, vegetative	Hexanal	MS/RI/O	5	3	2	5	5
1-12	orange	N-octanal	MS/RI/O	12	27	15	0	0.587
1-13	oily nutty	Nonanal	MS/RI/O	119	219	128	47	1.1
1-14	waxy, fatty	Decanal	MS/RI/O	10	11	7	9	3
1-15	rose	Benzaldehyde	MS/RI/O/ STD	0	0	1	1	750.89
1-16	honey, sweet	Phenylacetaldehyde	MS/RI/O/ STD	2	6	12	20	6.3
1-17	musty potato	3-Methylthioacetaldehyde	MS/RI/O	0	0	0	31	0.45
1-18	sour vinegar	Acetic acid	MS/RI/O	0	0	1	0	2200
1-19	sour fatty sweat cheese	Caproic acid	MS/RI/O	1	0	0	0	36
1-20	smoky flavor	2-Methoxyphenol	MS/RI/O/ STD	7	6	3	1	1.6
1-21	smoky flavor	4-Vinyl guaiacol	MS/RI/O	3	5	11	11	12.02
1-22	sweet creamy	2,3-Butyrolactone	MS/RI/O	49	1265	1874	442	0.059
1-23	popcorn	Acetoin	MS/RI/O	1	32	26	2	14
1-24	nutty	6-Methyl-2-ethylpyrazine	MS/RI/O/ STD	0	1	1	0	40
1-25	nutty	5-Methyl-2-ethylpyrazine	MS/RI/O	2	2	1	0	16
1-26	nutty	2,5-Dimethyl-3-ethylpyrazine	MS/RI/O	15	20	4	0	9
1-27	nutty	3,5-Dimethyl-2-ethylpyrazine	MS/RI/O	0	92	0	0	0.16
1-28	fruity green earthy	2-Pentylfuran	MS/RI/O	2	2	1	2	5.8
1-29	nutty and bready	2-Acetylpyridine	MS/RI/O/STD	0	0	0	1	19
1-30	sulfurous cabbage	Dimethyl disulfide	MS/RI/O	0	0	0	3	1.1
1-31	sweet, licorice, phenolic	4-Allyl benzyl ether	MS/RI/O	0	0	1	0	16

### Appendix A.3

**Table A2:** Key flavor compounds in koji room 2.

Number	Odor Characteristics	Compound	Testing Methods	ZQ0602	ZQ1202	ZQ2002	ZQ4002	Threshold (μg/L)
2-1	fruity	3-Methyl-1-butanol	MS/RI/O/ STD	0	0	1	1	220
2-2	green, fruity	1-Hexanol	MS/RI/O	2	1	1	0	5.6
2-3	mushroom	1-octen-3-ol	MS/RI/O/ STD	22	28	112	159	1.5
2-4	popcorn	Maltol	MS/RI/O/ STD	0	1	1	0	1240
2-5	sulfurous onion	3-Methylthiopropanol	MS/RI/O	0	0	1	1	123.23
2-6	fruity sweet	Acetic ether	MS/RI/O/ STD	11	12	5	5	5
2-7	fruity, creamy	Ethyl hexadecanoate	MS/RI/O	0	0	1	0	200
2-8	-	Methyl caprate	MS/RI	0	0	0	1	4.3
2-9	fruity	3-Methylbutyraldehyde	MS/RI/O	13	23	942	948	1.1
2-10	woody, vegetative	Hexanal	MS/RI/O	4	3	4	5	5
2-11	oily nutty	Nonana	MS/RI/O	129	126	116	56	1.1
2-12	waxy, fatty	Decanal	MS/RI/O	10	12	7	9	3
2-13	rose	Benzaldehyde	MS/RI/O/STD	0	0	1	1	750.89
2-14	honey, sweet	Phenylacetaldehyde	MS/RI/O/STD	3	4	22	17	6.3
2-15	musty potato	3-Methylthioacetaldehyde	MS/RI/O	0	0	29	53	0.45
2-16	orange	N-octanal	MS/RI/O	11	0	0	0	0.587
2-17	soapy, waxy, citrus	Dodecanal	MS/RI/O	0	9	0	0	0.29
2-18	sour fatty sweat cheese	Hexanoic acid	MS/RI/O	1	0	0	0	36
2-19	smoky flavor	2-Methoxyphenol	MS/RI/O/STD	6	8	5	7	1.6
2-20	smoky flavor	4-Vinyl guaiacol	MS/RI/O	3	5	6	6	12.02
2-21	popcorn	Acetoin	MS/RI/O	0	31	9	1	14
2-22	sweet creamy	2,3-Butyrolactone	MS/RI/O	45	1610	641	258	0.059
2-23	nutty	6-Methyl-2-ethylpyrazine	MS/RI/O/STD	0	0	1	0	40
2-24	nutty	5-Methyl-2-ethylpyrazine	MS/RI/O/STD	3	3	2	0	16
2-25	fruity green earthy	2-Pentylfuran	MS/RI/O	2	1	1	1	5.8
2-26	sulfurous cabbage	Dimethyl disulfide	MS/RI/O	0	0	0	9	1.1
2-27	sulfurous, alliaceous	Dimethyl trisulfide	MS/RI/O	0	0	0	32	0.1

### Appendix A.4

**Table A3:** Key flavor compounds in koji room 3.

Number	Odor Characteristics	Compound	Testing Methods	ZQ0603	ZQ1203	ZQ2003	ZQ4003	Threshold (μg/L)
3-1	fruity	3-Methyl-1-butanol	MS/RI/O/STD	0	0	1	2	220
3-2	green, fruity	1-Hexanol	MS/RI/O	3	2	2	0	5.6
3-3	mushroom	1-octen-3-ol	MS/RI/O/STD	28	48	66	131	1.5
3-4	sulfurous onion	3-Methylthiopropanol	MS/RI/O	0	0	1	2	123.23
3-5	fruity sweet	Acetic ether	MS/RI/O/STD	14	8	4	0	5
3-6	-	2-Ethylhexyl acrylate	MS/RI	4	4	0	0	5
3-7	fruity	3-Methylbutyraldehyde	MS/RI/O	18	33	358	702	1.1
3-8	woody, vegetative	Hexanal	MS/RI/O	4	3	5	4	5
3-9	oily nutty	Nonanal	MS/RI/O	101	98	74	50	1.1
3-10	waxy, fatty	Decanal	MS/RI/O	13	11	8	12	3
3-11	rose	Benzaldehyde	MS/RI/O/ STD	0	0	1	0	750.89
3-12	honey, sweet	Phenylacetaldehyde	MS/RI/O/ STD	2	6	12	13	6.3
3-13	musty potato	3-Methylthioacetaldehyde	MS/RI/O	0	0	34	39	0.45
3-14	soapy, waxy, citrus	Dodecanal	MS/RI/O	0	12	0	0	0.29
3-15	orange	N-octanal	MS/RI/O	0	0	7	0	0.587
3-16	sour fatty sweat cheese	Hexanoic acid	MS/RI/O	1	0	0	0	36
3-17	smoky flavor	2-Methoxyphenol	MS/RI/O/STD	12	7	4	0	1.6
3-18	smoky flavor	4-Vinyl guaiacol	MS/RI/O	4	4	4	3	12.02
3-19	sweet creamy	2,3-Butyrolactone	MS/RI/O	64	2250	1273	261	0.059
3-20	popcorn	Acetoin	MS/RI/O	0	20	10	2	14
3-21	creamy cheese	2-Ketocholesterol	MS/RI/O	0	0	0	1	5.5
3-22	nutty	5-Methyl-2-ethylpyrazine	MS/RI/O	3	3	1	0	16
3-23	nutty	2,5-Dimethyl-3-ethylpyrazine	MS/RI/O	26	24	3	0	9
3-24	nutty	3,5-Dimethyl-2-ethylpyrazine	MS/RI/O	6	0	0	0	2
3-25	fruity green earthy	2-Pentylfuran	MS/RI/O/ STD	1	1	2	1	5.8

Note: Identification methods for each odor compound: MS, RI, O, and STD denote identification via mass spectrometry, retention index, olfactory analysis, and standard compounds, respectively; the perceived odor of each substance detected by the olfactory port; The matrix used to determine each threshold was water, as described in the book *Odor Thresholds* (a compilation of odor thresholds in air, water, and other media).

### Appendix A.5

**Table A4:** Free amino acid content during koji fermentation.

Name	ZQ0601	ZQ1201	ZQ2001	ZQ4001	ZQ0602	ZQ1202	ZQ2002	ZQ4002	ZQ0603	ZQ1203	ZQ2003	ZQ4003
Asp	0.05 ± 0.01	0.27 ± 0.00	0.43 ± 0.00	2.36 ± 0.00	0.04 ± 0.00	0.23 ± 0.00	0.60 ± 0.01	2.78 ± 0.02	0.06 ± 0.00	0.25 ± 0.00	0.35 ± 0.00	1.64 ± 0.03
Glu	0.08 ± 0.00	0.27 ± 0.00	0.87 ± 0.01	4.58 ± 0.05	0.05 ± 0.00	0.21 ± 0.00	1.65 ± 0.02	4.66 ± 0.02	0.09 ± 0.01	0.31 ± 0.00	0.75 ± 0.01	4.09 ± 0.10
Ser	0.01 ± 0.01	0.09 ± 0.01	0.41 ± 0.00	1.31 ± 0.00	0.01 ± 0.00	0.06 ± 0.00	0.40 ± 0.00	1.68 ± 0.01	0.01 ± 0.00	0.14 ± 0.00	0.32 ± 0.00	1.02 ± 0.01
His	0.03 ± 0.00	0.13 ± 0.01	0.21 ± 0.00	0.47 ± 0.01	0.03 ± 0.00	0.13 ± 0.01	0.24 ± 0.03	0.46 ± 0.01	0.04 ± 0.00	0.15 ± 0.01	0.18 ± 0.01	0.24 ± 0.02
Gly	0.01 ± 0.00	0.04 ± 0.01	0.15 ± 0.00	0.75 ± 0.05	0.01 ± 0.00	0.03 ± 0.00	0.18 ± 0.01	0.83 ± 0.01	0.01 ± 0.01	0.06 ± 0.00	0.12 ± 0.00	0.53 ± 0.01
Thr	0.08 ± 0.01	0.16 ± 0.01	0.18 ± 0.01	0.99 ± 0.00	0.07 ± 0.00	0.11 ± 0.00	0.27 ± 0.04	1.22 ± 0.01	0.08 ± 0.01	0.15 ± 0.00	0.21 ± 0.00	0.64 ± 0.02
Arg	0.44 ± 0.02	0.82 ± 0.02	0.36 ± 0.01	1.04 ± 0.01	0.30 ± 0.00	0.69 ± 0.01	0.53 ± 0.00	1.46 ± 0.02	0.47 ± 0.00	0.71 ± 0.01	0.30 ± 0.01	0.77 ± 0.02
Ala	0.08 ± 0.04	0.24 ± 0.01	0.29 ± 0.01	1.17 ± 0.00	0.03 ± 0.00	0.16 ± 0.00	0.28 ± 0.00	0.78 ± 0.01	0.06 ± 0.01	0.39 ± 0.00	0.19 ± 0.00	0.65 ± 0.02
Tyr	0.10 ± 0.01	0.20 ± 0.01	0.26 ± 0.04	0.53 ± 0.00	0.07 ± 0.00	0.17 ± 0.01	0.29 ± 0.04	0.75 ± 0.01	0.10 ± 0.01	0.24 ± 0.01	0.21 ± 0.01	0.41 ± 0.01
Cys	0.02 ± 0.01	0.04 ± 0.00	0.03 ± 0.00	0.14 ± 0.00	0.02 ± 0.00	0.03 ± 0.00	0.04 ± 0.01	0.12 ± 0.00	0.03 ± 0.00	0.04 ± 0.00	0.03 ± 0.01	0.08 ± 0.01
Val	0.01 ± 0.01	0.07 ± 0.00	0.06 ± 0.00	1.01 ± 0.00	0 ± 0.003	0.05 ± 0.00	0.30 ± 0.00	1.21 ± 0.01	0.01 ± 0.01	0.1 ± 0.01	0.20 ± 0.02	0.73 ± 0.00
Met	0.05 ± 0.01	0.07 ± 0.01	0.07 ± 0.01	0.41 ± 0.00	0.04 ± 0.00	0.06 ± 0.00	0.11 ± 0.01	0.53 ± 0.01	0.06 ± 0.00	0.08 ± 0.01	0.1 ± 0.01	0.31 ± 0.00
Phe	0.01 ± 0.01	0.11 ± 0.00	0.11 ± 0.00	1.24 ± 0.01	0.01 ± 0.00	0.08 ± 0.01	0.44 ± 0.00	1.33 ± 0.01	0.01 ± 0.01	0.15 ± 0.02	0.27 ± 0.01	0.78 ± 0.02
Ile	0.01 ± 0.00	0.04 ± 0.01	0.26 ± 0.01	1.09 ± 0.00	0.01 ± 0.00	0.03 ± 0.00	0.34 ± 0.00	0.86 ± 0.01	0.01 ± 0.01	0.06 ± 0.01	0.13 ± 0.01	0.51 ± 0.01
Leu	0.00 ± 0.00	0.11 ± 0.00	0.47 ± 0.00	1.78 ± 0.02	0.01 ± 0.00	0.09 ± 0.01	0.60 ± 0.03	2.31 ± 0.02	0.00 ± 0.00	0.16 ± 0.00	0.4 ± 0.01	1.43 ± 0.03
Lys	0.04 ± 0.01	0.18 ± 0.00	0.38 ± 0.05	1.35 ± 0.02	0.03 ± 0.00	0.12 ± 0.00	0.52 ± 0.01	1.76 ± 0.01	0.04 ± 0.00	0.29 ± 0.00	0.28 ± 0.01	1.00 ± 0.01

Note: Data are presented as the mean ± standard deviation (SD) of three technical replicates.
